# Differential sRNA Regulation in Leaves and Roots of Sugarcane under Water Depletion

**DOI:** 10.1371/journal.pone.0093822

**Published:** 2014-04-02

**Authors:** Flávia Thiebaut, Clícia Grativol, Milos Tanurdzic, Mariana Carnavale-Bottino, Tauan Vieira, Mariana Romeiro Motta, Cristian Rojas, Renato Vincentini, Sabrina Moutinho Chabregas, Adriana Silva Hemerly, Robert A. Martienssen, Paulo Cavalcanti Gomes Ferreira

**Affiliations:** 1 Laboratório de Biologia Molecular de Plantas, Instituto de Bioquímica Médica, Universidade Federal do Rio de Janeiro, Rio de Janeiro, Rio de Janeiro, Brazil; 2 School of Biological Sciences, The University of Queensland, Brisbane St Lucia, Queensland, Australia; 3 Universidade Federal da INTEGRAÇÃO Latino-Americana, Foz do Iguaçu, Paraná, Brazil; 4 Centro de Biologia Molecular e Engenharia Genética, Universidade Estadual de Campinas, Campinas, São Paulo, Brazil; 5 Centro de Tecnologia Canavieira, Fazenda Santo Antônio – Laboratório de Biologia Molecular, Piracicaba, São Paulo, Brazil; 6 Howard Hughes Medical Institute and Gordon and Betty Moore Foundation, Cold Spring Harbor Laboratory, Cold Spring Harbor, New York, United States of America; East Carolina University, United States of America

## Abstract

Plants have developed multiple regulatory mechanisms to respond and adapt to stress. Drought stress is one of the major constraints to agricultural productivity worldwide and recent reports have highlighted the importance of plant sRNA in the response and adaptation to water availability. In order to increase our understanding of the roles of sRNA in response to water depletion, cultivars of sugarcane were submitted to treatment of ceasing drip irrigation for 24 hours. Deep sequencing analysis was carried out to identify the sRNA regulated in leaves and roots of sugarcane cultivars with different drought sensitivities. The pool of sRNA selected allowed the analysis of different sRNA classes (miRNA and siRNA). Twenty-eight and 36 families of conserved miRNA were identified in leaf and root libraries, respectively. Dynamic regulation of miRNA was observed and the expression profiles of eight miRNA were verified in leaf samples from three biological replicates by stem-loop qRT-PCR assay using the cultivars: SP90–1638 - sensitive cultivar- and; SP83–2847 and SP83–5073 - tolerant cultivars. Altered miRNA regulation was correlated with changes in mRNA levels of specific targets. Two leaf libraries from individual sugarcane cultivars with contrasting drought-tolerance properties were also analyzed. An enrichment of 22-nt sRNA species was observed in leaf libraries. 22-nt miRNA triggered siRNA production by cleavage of their targets in response to water depletion. A number of genes of the sRNA biogenesis pathway were down-regulated in tolerant genotypes and up-regulated in sensitive in response to water depletion treatment. Our analysis contributes to increase the knowledge on the roles of sRNA in sugarcane submitted to water depletion.

## Introduction

Epigenetic regulation has gained increasing interest recently as a possible source of variation for plant breeding [1]. Several studies have shown that epigenetic regulation is essential for plant developmental processes [2], modifying our understanding of how plants can respond and adapt to different stress situations [3], [4]. Epigenetic regulation is coupled with small RNA (sRNA) to bring about transcriptional gene silencing via DNA and histone methylation, or can act at post-transcriptional level by targeting mRNA for degradation via microRNA (miRNA)-directed cleavage [5]. sRNA are increasingly being investigated as an epigenetic mechanism involved in responses to stresses in plants [6].

The discovery of small non-coding RNA with regulatory functions has modified our notion of gene, both in terms of its definition and gene regulation. These small RNA provide regulatory plasticity, and are divided into two categories: microRNA (miRNA) and small interfering RNA (siRNA), which include *trans*-acting small interfering RNA (ta-siRNA) [7]. These sRNA classes are generated by the processing of a double-stranded RNA (dsRNA) precursor by RNase III-like Dicer proteins (DCL) [8]. However, the type of precursor, the enzymes of the biogenesis pathways, and mechanism of action can distinguish the sRNA classes.

The canonical miRNA are endogenous 21–24-nucleotide (nt) long sequences transcribed from MIR genes [9]. In plants, the primary transcripts of MIR genes are stem-loop partially double-stranded RNA structures, which are processed by DCL1 and two other proteins, HYPONASTIC LEAVES 1 (HYL1) and SERRATE (SE) to generate a miRNA/miRNA* duplex, which is methylated in both strands by HUA ENHANCER 1 (HEN1) [10] and exported by HASTY (HST) from the nucleus to the cytoplasm [11]. Next, one strand, the mature miRNA, is loaded into the RNA-Induced Silencing Complex (RISC), a ribonucleoprotein complex which includes the ARGONAUTE1 (AGO1) protein [12]. AGO1 then binds the target mRNA transcript via complementarity to the miRNA sequence previously loaded in the active RISC. In plants, the regulation of expression occurs by target mRNA cleavage with near-perfect complementarity, or, less frequently, by translational inhibition or DNA methylation [13], [14].

Precursors of siRNA, in contrast, are originated mainly from transcripts from repetitive DNA in the genome, but also by exogenous viral sources [15]. These transcripts are then made double stranded via the action of an RNA Dependent RNA polymerase activity (mainly RDR2) and further processed into siRNA by DCL3 [16] which, similarly to miRNA, are loaded in AGO4, and participate in directing DNA and histone methylation to the target locus, thereby causing its transcriptional silencing [17].

Trans-acting siRNA, on the other hand, are reminiscent of miRNA in terms of their role in post-transcriptional gene regulation; however, their biogenesis shares common processes with miRNA and siRNA. The primary ta-siRNA (pri-ta-siRNA) is a single-stranded RNA, which contains a target site for miRNA binding, necessary to trigger the ta-siRNA formation. After the cleavage of the pri-ta-siRNA, RNA-dependent RNA polymerase 6 (RDR6) generates a double-strand from 3′ fragment, the ta-siRNA precursor (pre-ta-siRNA). This precursor is processed by DCL4 into a duplex that will originate 21-nt ta-siRNA [18]. Remarkably, ta-siRNA mediated regulation is an ancient pathway, once they occur in mosses and all the way to flowering plants [19], [20].

An important area of investigation is the role of sRNA regulation upon environmental stresses [21]. Given that RNAi mechanisms have been retained in all major plant clades, it is not surprising that miRNA and different classes of siRNA have been reported to participate in plant responses to abiotic stress [22]. A large fraction of the miRNA families, the most abundant in the majority of the plants, was first described in model species, such as *Arabidopsis thaliana*, where almost the entire genome sequence is available [5], [23]. However, in polyploid plants with large genomes, where a complete genomic sequence is still missing, such as sugarcane (*Saccharum spp.*); miRNA discovery is significantly more complex. On the other hand, high-throughput sequencing technologies and bioinformatics analyzes enable the identification of conserved microRNA sequences by comparison with databases of small RNA sequences [24], [25].

Sugarcane is a monocot plant from the Poaceae family, whose production is responsible for 60% of the raw sugar manufactured worldwide; it also represents an important source of biofuel. Despite of its economic importance, sugarcane gene regulatory pathways have not been studied extensively, in part due to its genomic complexity [26]. The description of gene regulatory pathways in sugarcane is an important step in a possible creation of tolerant varieties to drought, which is major goal in countries where sugarcane is cultivated.

As a first step to investigate the role sRNA regulation in the response of sugarcane to water depletion, we constructed and sequenced sRNA libraries from different cultivars of sugarcane in control and stress situation. Bioinformatics analyses identified 28 and 36 conserved miRNA families in leaf and root libraries, respectively. Two leaf libraries from individual sugarcane cultivars that have contrasting to drought-tolerance properties were also analyzed. MiRNA are differentially expressed in leaves and roots upon treatment with water depletion. The expression profiles of eight miRNA (miR156, miR159 (two isoforms), miR164, miR167, miR168, miR169 and miR397) differentially expressed in leaves were examined using stem–loop RT-PCR assay. The biological functional of miRNA was accessed by analyzing their putative targets. Furthermore, a large-scale investigation of sRNA in sugarcane using a computational approach has identified siRNA candidates from targets of miRNA with 22-nt in the length. The expression of one 21 nt siRNA, which is present in all libraries, was up-regulated in three different cultivars in response to water depletion. To investigate the distribution of the siRNA fraction was aligned in cluster of sugarcane repeats, and the T0h libraries have shown a distinct distribution of siRNAs. Furthermore, expression analyzes of genes from the machinery of sRNA biogenesis, including DCL1, DCL2, RDR6, HYL1, and three genes encoding of AGO1 and AGO4 have shown down-regulation in drought tolerant cultivars and up-regulation in sensitive cultivar in response to 24 h of water depletion. Our findings contribute to increase the knowledge of sRNA roles in response to water depletion in the complex, polyploidy sugarcane plant.

## Materials and Methods

### Plant Material and Experimental Procedure

The Centro de Tecnologia Canavieira (CTC) provided stalks of sugarcane cultivars, with different drought sensitivities. Based on chlorophyll and water content measurements, cultivars CTC15, CTC6, SP83–2847 and SP83–5073, and CTC9, CTC13, SP90–1638 and SP90–3414 were considered as drought tolerant and sensitive, respectively. Stalks were planted germinated and grown in 5 L pots with a mixture of soil and sand (2∶1) in a greenhouse at 28°C, and irrigated by dripping. After three months, the plants were exposed to drought by withholding water for 24 hours. Treated and control roots and shoots were harvested at 0 and 24 hours of treatment, respectively. Preparation of small RNA libraries of root samples was described previously [27]. Similarly, four leaf sRNA libraries were constructed from RNA pools from sensitive and tolerant sugarcane cultivars submitted to water depletion or from control plants. Additionally, three experiments were carried out using the same experimental procedure to obtain plant material to validate the bioinformatics analysis of the libraries. However, for this experiment only three cultivars (SP83–2847 and SP83–5073 and SP90–1638) were used. Besides, only shoots were harvested.

### RNA Extraction and Sequencing Small RNA Library Construction

Sensitive and tolerant cultivars in each experiment were grouped as a pool of treated plants (plants that received the treatment of water depletion) and control plants. Total RNA for each pool was isolated from fresh root and leaf plant material using Trizol (Invitrogen, CA, USA) as described by the manufacturer. The amount of RNA was measured using Thermo Scientific NanoDrop 2000c Spectrophotometer and then the quality was verified by electrophoresis on a 1% agarose gel. The RNA was used to construct the small RNA libraries, using Illumina-based small RNA cloning protocol [28]. Detailed protocol is available upon request. In addition, using the same strategy cited above, more two sRNAs libraries were constructed using leaves from individual cultivar one tolerant cultivar (CTC6) and one sensitive cultivar (SP903414) submitted to 24 h of water deficit. After this, sRNAs were sequenced on the Illumina GAII sequencer at Cold Spring Harbor Laboratory. The sequence data from this study have been submitted to NCBI - Gene Expression Omnibus (http://www.ncbi.nlm.nih.gov/geo/) under accession number GSE42483. The quality of the sequences was evaluated by measuring the quality of the reads according to the percentage of bases having a base quality greater or equal than 30 (Q30). On average, 80% of each flow cell lane had Q30 of quality. Next, adapters and “N” bases were trimmed of the reads, and reads in the 18–28 nt range were separated for further analysis. Using the UEA sRNA toolkit-Plant version filter pipeline (http://srna-tools.cmp.uea.ac.uk/) [29], and three different databases - all RNA from Rfam, all Arabidopsis tRNA from The Genomic tRNA Database and all plant t/rRNA sequences from EMBL release - reads with low-complexity (less than 3 different bases) and both sense and antisense matches with different types of RNA (e.g. sno/t/rRNA) were removed.

### Bioinformatics Analysis

#### Identification and electronic northern of miRNA

Filtered sRNAs reads were submitted to the University of East Anglia sRNA toolkit-Plant version miRProf, which determines the expression levels of sRNAs matching them to known miRNAs at miRBase and at a specific genome. The expression level of a miRNA represents the number of occurrences of the sequence in the library. miRProf was run with sRNA minimum size of 18 nt, maximum size of 28 nt. The sRNA libraries were matched to known Viridiplantae mature miRNA deposited in the miRBase database release 17 (http://www.mirbase.org/ftp.shtml) and to *Sorghum bicolor* genome (JGI, v1.0) by using PatMaN (a miRProf inside program). The output of the miRProf showed sequences of miRNA that had a maximum of three mismatches with the miRBase and sorghum genome and their unique sequence counts. To allow comparison between libraries, unique counts were normalized in reads *per* 1 million (RPM) and the total number of filtered reads for each library was used for normalization. With the identified miRNA sequences and their normalized expression numbers, we represented the miRNA expression levels on each sample as electronic northern. The changes of miRNA expressions between libraries were evaluated by using the IDEG6 software available at (http://telethon.bio.unipd.it/bioinfo/IDEG6_form/). The statistical analysis (Fisher exact test) was performed with a p-value cutoff <0.05 and Bonferroni correction. The normalized miRNA expression data have been submitted to NCBI - Gene Expression Omnibus (http://www.ncbi.nlm.nih.gov/geo/) under accession number GSE42483.

MiRNAs from sRNAs libraries of CTC6 (tolerant cultivar) and SP903414 (sensitive cultivar) submitted to water depletion (24 h) were identified by a similar pipeline. The following comparisons of miRNA profile among pool libraries and individual cultivar libraries were performed: Comparison 1– influence of genetic variation on miRNA response to water depletion (T24h/S24h vs. CTC6/SP903414); Comparison 2– influence of water depletion treatment in miRNA response on each genotype (CTC6/T0h vs. T24h/T0h) and (SP903414/S0h vs. S24h/S0h).

#### miRNA target prediction

To identify putative miRNA targets, we used the standalone-based UEA sRNA toolkit-Plant target prediction pipeline. This tool allows choosing transcript databases for searching targets. In this investigation, we have used *Saccharum officinarum* ESTs – DFCI gene Index release 3. MiRNA/target duplexes must obey the following rules: i) no more than four mismatches between sRNA and target (G-U bases count as 0.5 mismatches); ii) no more than two adjacent mismatches in the miRNA/target duplex; iii) no adjacent mismatches in positions 2–12 of the miRNA/target duplex (5′ of miRNA); iii) no mismatches in positions 10–11 of miRNA/target duplex; no more than 2.5 mismatches in positions 1–12 of the of the miRNA/target duplex; iv) and the Minimum Free Energy (MFE) of the miRNA/target duplex should be> = 74% of the MFE of the miRNA bound to its perfect complement.

#### Analysis of siRNA candidates

Analysis of size distribution of reads unraveled a large fraction of sRNA of 22-nts, among the sRNA. Putative targets of these 22 nt sRNA were selected to identify putative ta-siRNA candidates. These targets were aligned to sRNA libraries without the previously identified miRNA sequences, using BLASTN tools with no gaps and minimum alignment of 18 bases.

To classify the fraction of sRNA present in all libraries, we aligned the sRNA present in all libraries, excluding the list of miRNA, in clusters of siRNA. The clusters were constructed with a dataset of small RNA libraries (approximately 20 libraries, with miRNA sequences excluded) and the annotation was based s in the classification of a sugarcane repeat database (Grativol *et* al., in preparation) and in the sugarcane EST database.

### Expression Analysis by qRT-PCR

The expression profiles of eight sugarcane mature miRNA (miR156, miR159 (two isoforms), miR164, miR167, miR168, miR169 and miR397) and ta-siRNA candidates were assayed by stem–loop reverse transcription-PCR [30], [31]. Total RNA extracted from leaves used in the small RNA libraries construction was treated with DNaseI (Promega). Total RNA was then reverse transcribed into cDNA using Super-Script III reverse transcriptase (Invitrogen). In the same reaction, RT primers specific of each sRNA and random primers were used to enable the amplification of the constitutively expressed control gene and the miRNA targets. Moreover, the expression profile of the genes involved in the synthesis of sRNA (DCL1, DCL2, RDR6, HYL1, AGO1 and AGO4) was investigated by qRT-PCR with samples obtained from sugarcane plants obtained using the same experimental conditions and the same cDNA described above.

Using this cDNA as template, qRT-PCR was done with SYBR Green PCR Master Mix (Applied Biosystems) using: 1 μL of first strand cDNA, 5 μL of SYBR Green solution, 2 μL of the forward primer (10 μM) and 2 μL of reverse primer (10 μM) [30]. Glyceraldehyde-3-phosphate dehydrogenase - GAPDH (CA254672.1 - F/5′CACGGCCACTGGAAGCA3′, R/5′TCCTCAGGGTTCCTGATGCC3′) and 28S (F/5′GCGAAGCCAGAGGAAACT3′, R5′GACGAACGATTTGCACGTC3′) were used as the internal control. GAPDH and 28S were validated as sugarcane housekeeping genes in different conditions [32]–[35]. qRT-PCR was performed using an Applied Biosystems 7500 Real-Time PCR Systems. To confirm the expression of miRNA and their targets three biological replicates were analyzed.

## Results

### Enrichment of 22-nt sRNA Species is Tissue Specific

**Figure 1:**
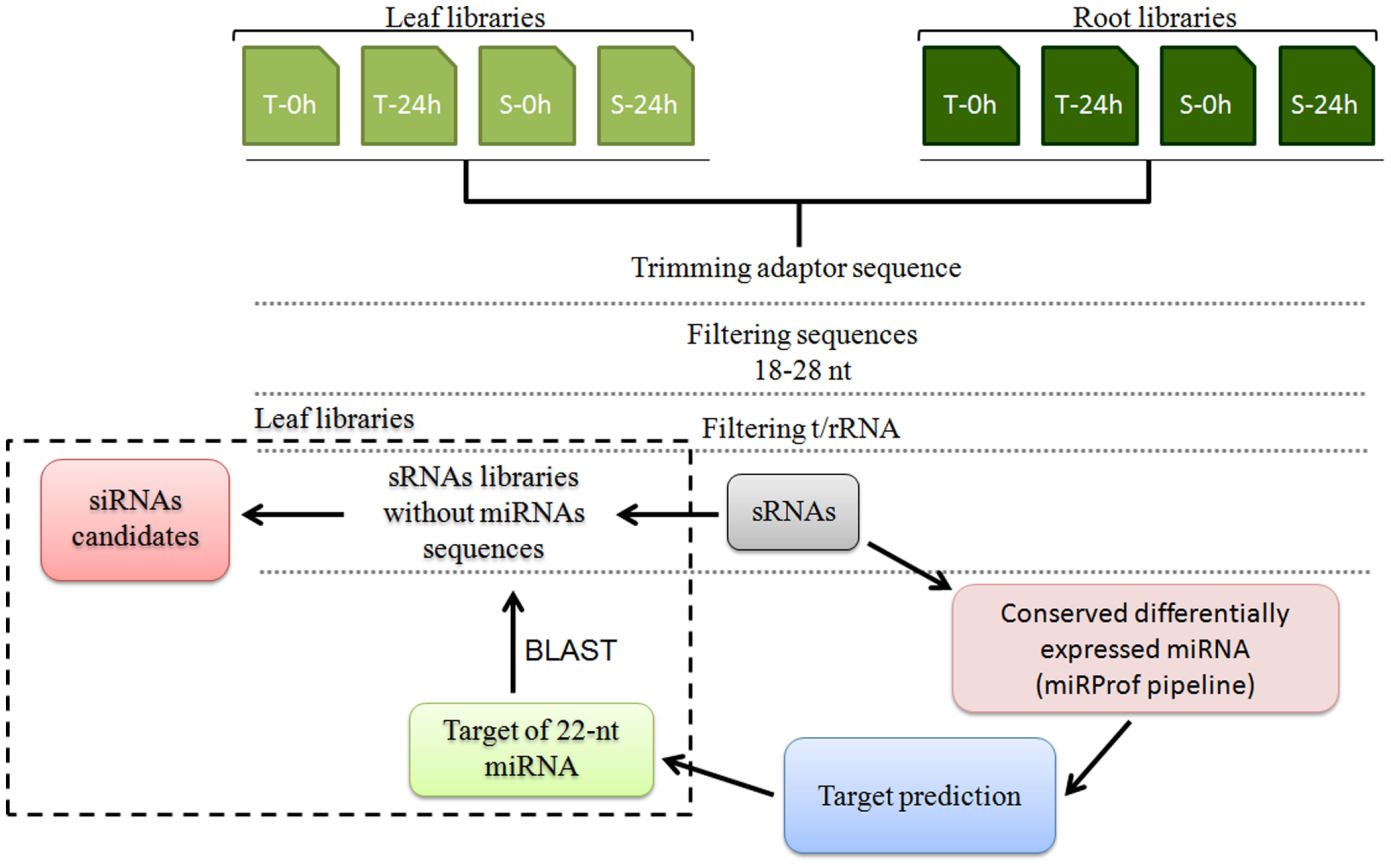
Pipeline for analysis of sRNA libraries. Pipeline used to identify sugarcane sRNA regulated by water depletion from leaf and root sRNA libraries. The identification of siRNA candidate (dotted box) was performed only in leaf libraries. T = tolerant sugarcane cultivar, S = sensitive sugarcane cultivar. 0 h = control of stress, 24 h = time of water depletion treatment.

**Figure 2:**
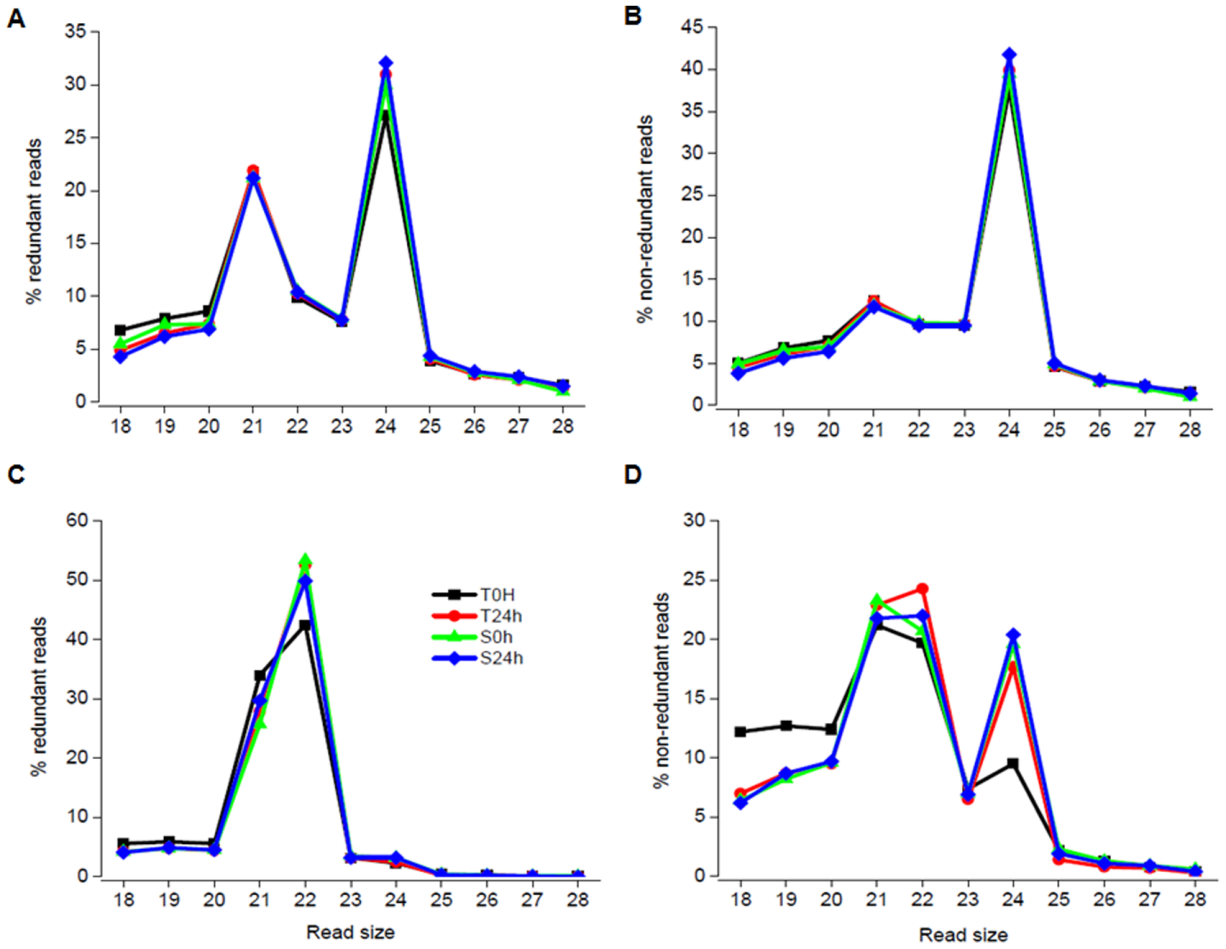
Small RNA reads sizes distribution. Graphs depict the length distribution of redundant and non-redundant small RNA dataset in root (A and B) and in leaf (C and D). T = tolerant sugarcane cultivar, S = sensitive sugarcane cultivar.

Eight sRNA libraries were constructed and sequenced (four from leaf RNA and four from root RNA) to explore the role of sRNA in sugarcane plants under water depletion. In each case, we have used RNA isolated from pools of sugarcane cultivars submitted to water depletion for 24 hours and control plants. These cultivars were previously classified as tolerant or sensitive to water depletion according to sugarcane production, chlorophyll content and water content under drought stress (data not shown). Illumina-based sequencing yielded approximately 35 million and 24 million reads in leaf and root libraries, respectively (Table 1). To analyze the sRNA population, we followed the bioinformatics pipeline described in the Figure 1 and methods. Approximately 14 million filtered reads in leaves - corresponding to 164,422 unique sequences; and approximately 16 million in roots – were obtained, corresponding to 1,210,502 unique sequences (Table 1). After trimming and filtering adaptors and tRNA/rRNA sequences, the remaining sRNA sequences were analyzed for size distribution (Figure 2). In all root libraries, the most abundant sRNAs were the 21 and 24 nucleotide (nt) in length, respectively (Figure 2A). The more complex fraction was the one containing the 24 nt sRNA (Figure 2B). However, in leaf libraries an additional peak of 22 nt was observed in the distribution of redundant reads (Figure 2C, D). Accordingly, the distribution of leaf non-redundant reads also showed three peaks, 21 nt, 22 nt and 24 nt.

**Table 1:** Summary of results obtained after computational data mining of small RNA libraries.

Description	Leaf libraries	Root libraries
**Total**		
All reads	35,755,991	24,243,352
Filtered reads^a^	14,193,780	16,956,062
**Unique**		
Filtered reads^a^	164,422	1,210,502
Conserved sRNA^b^	88	273

a Filtering for tRNA, rRNA, low-complexity sequence and trimming for “N” bases and 3′ adapters.

b Conserved miRNA deposited at miRBase database identified by miRProf pipeline.

### The miRNA set is more Complex in Root Libraries

**Figure 3:**
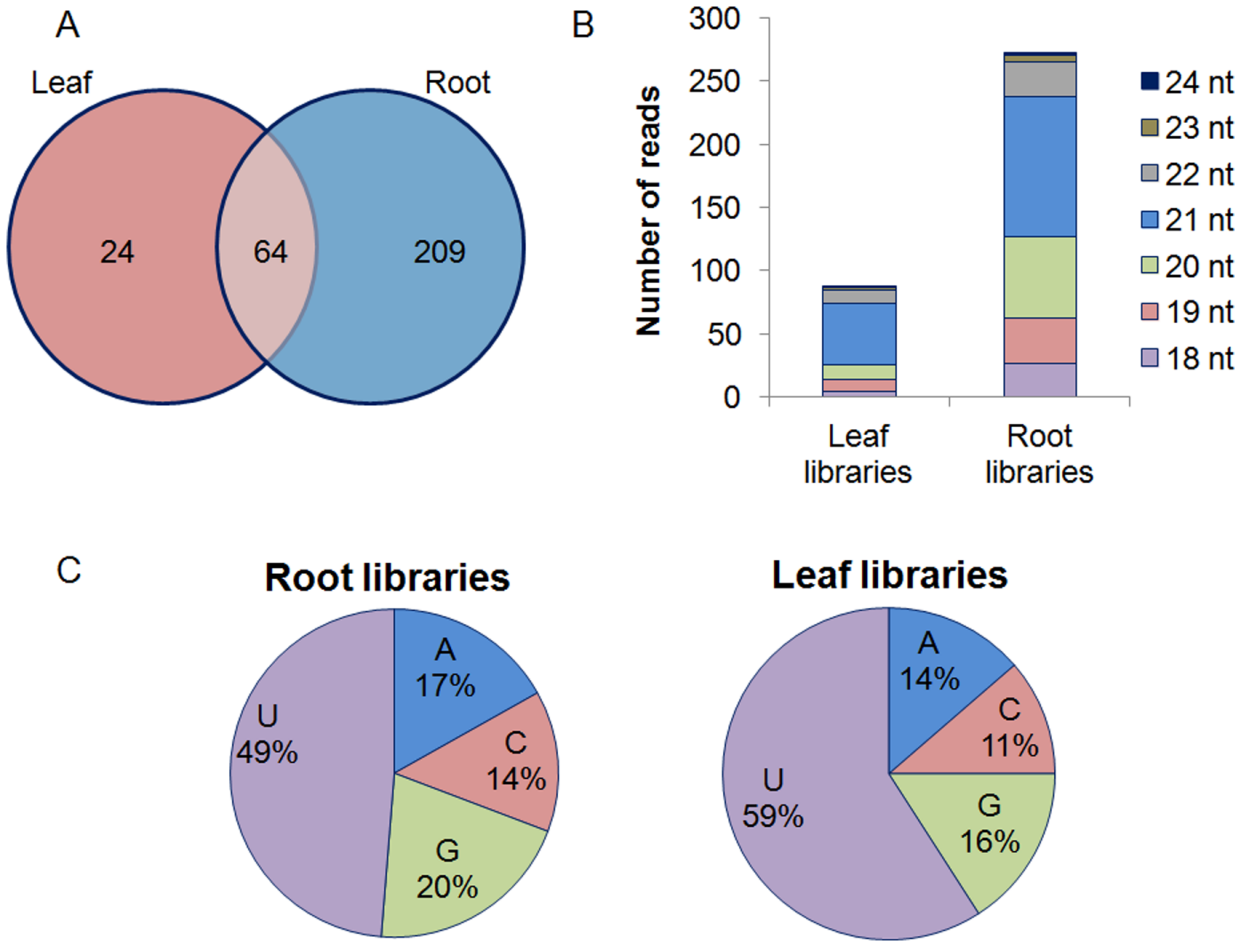
Distribution of conserved miRNA identified. (A) Diagram shows number of miRNA in common and unique in leaf and root libraries. (B) Distribution of miRNA length in leaf and root libraries. (C) Percentage of miRNA identified based on 5′ terminus nucleotide. A: adenine; U: uracile (thymine conversion); C: cytosine; G: guanine.

In order to identify the sugarcane miRNAs, the set of sRNAs recovered from the libraries were aligned to the miRBase database, accepting up to three mismatches. A total of 205 and 312 unique miRNA sequences were found in leaf and root libraries, respectively. Out of these, 88 and 273 (leaf and root, respectively) are conserved miRNA or miRNA* (Table 1), previously identified in other plants. The remaining sequences were classified as new miRNA candidates and their characterization has been described before [27]. Sixty-four sequences are common among libraries from the two tissues (Figure 3A). Overall, the identified conserved-miRNA corresponded to 28 miRNA families in leaves, and 36 miRNA families in roots. Eleven different miRNA* were found in the leaf libraries and 54 in the root libraries. The distribution of miRNA according to length in root and leaf libraries is shown in the figure 3B. The majority of the conserved miRNA had 21-nt in both tissues and the majority of miRNA had Uracil (U) at the 5′-end (Figure 3C).

### Dynamic Regulation of miRNA in Roots and Leaves under Water Depletion

The abundance of the different miRNAs can be calculated from their frequency in each library. To compare the abundance distribution of miRNAs in libraries from control samples and samples treated with water depletion, we normalized the miRNA reads and compared their detectable expression levels on each sample through electronic northern (Materials and Methods). Twenty known miRNA - from 88 identified in leaves - were expressed in all leaf libraries and 169 - from 273 were shared between root libraries (File S1 and S2, respectively). In the control, 94% of miRNA from leaf libraries have distinct expression patterns among cultivars (T0h and S0h). Among tolerant and sensitive cultivars submitted to water depletion (T24h and S24h), the percentage of miRNA differentially expressed (p<0.05) was reduced to 83% (File S3). A similar comparison was made in root samples; and 59% of miRNAs were differentially expressed in the cultivars, while 56% changed their expression in stressed plants (File S4). Independently of the condition, most miRNA were differentially expressed in libraries prepared from drought sensitive cultivars.

In order to evaluate if the miRNA profiles observed in pooled samples (tolerant and sensitive libraries) were replicated in individual genotypes, eight mature miRNA, identified in both tissues, were selected and their expression profile was analyzed by qRT-PCR using leaf samples from three biological replicates. In each experiment, leaves of the cultivars collected after 24 h of water depletion and control plants were used for this analysis. The selection of the cultivars was based on results of experiments performed in Centro de Tecnologia Canavieira – CTC, which evaluated of drought stress responses of 20 commercial sugarcane cultivars (Sabrina M. Chabregas, personal communication). The measurement was carried out after cessation of irrigation by dripping. The physiological parameters analyzed - chlorophyll content, fluorescence and relative water content - established different levels of drought tolerance among sugarcane cultivars, although the mechanisms triggered by the stress can vary, including leaf curling, stomata closure and cell wall thickening. Based on these parameters, the cultivars SP901638 was considered the most sensitive cultivar, while SP832847 and SP835073 were considered as tolerant cultivars. These two tolerant cultivars have important characteristics: SP832847 is distinguished by its high productivity, while SP835073 has elevated sucrose and fiber content.

**Figure 4:**
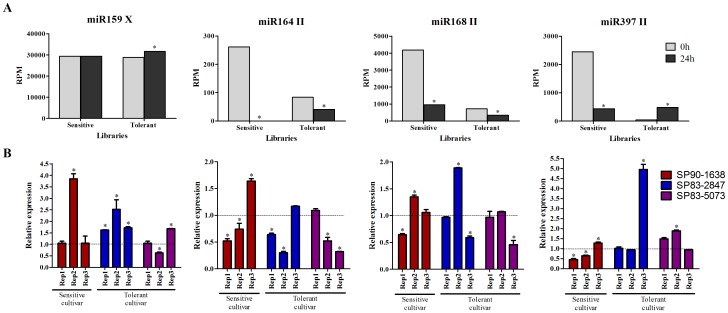
Comparison of miRNA expression profile. The expression profile of four miRNA were analyzed: miR159 X, miR164 II, miR168 II and miR397 II. (A) The graphs show the expression profile of miRNA based in bioinformatics analysis for each library (reads *per* million - RPM). *represent significantly changing of miRNA expression between control and treatment libraries (p-value <0.05). (B) The graphs show the relative expression of miRNA in each cultivar by qRT-PCR in three biological replicates. Three cultivars were used in these analyses (SP90–1638– sensitive cultivar; SP83–2847 and SP83–5073– tolerant cultivars). In each case, control condition had relative expression equal 1 (dotted line). *represent significantly changing of miRNA expression between control and treatment samples (p-value <0.05).

In four analyzed miRNAs (miR159X, miR168II, miR397II and miR164II), the differential expression profiles obtained by sequencing were similar with the results of the validation by RT-qPCR in at least one of biological replicates. Figure 4A shows the number of reads *per* million found in each leaf library for these miRNAs. These graphs allow the comparison of the abundance of these miRNA differentially expressed in response to the water depletion treatment. Similar profiles between the relative expressions obtained by pulsed RT-qPCR and bioinformatics-based expression analysis were observed for some, but not all, microRNAs. For the validation by RT-qPCR, samples from three experiments were used (Rep1, Rep2 and Rep3) to analyze the expression profile of the miRNAs in the cultivars SP901638, SP832847 and SP835073 (Figure 4B). In the sequencing analysis, miR159 was induced in tolerant cultivars submitted to water depletion (Figure 4A), and this expression profile was confirmed by RT-qPCR using the cultivar SP832847. However, the other tolerant cultivar showed different miR159 expression profiles between replicates samples, although in the Rep3, this miRNA was up-regulated (Figure 4B). In the sensitive cultivar its expression did not vary in bioinformatics analysis and in RT-qPCR analysis for Rep1 and Rep3 samples, while for Rep2 samples, miR159 was induced in response to water depletion (p-value <0.05). MiR164 II was significantly down-regulated in sRNA libraries of sensitive and tolerant cultivars. In two biological replicates this miRNAs was repressed in response to water depletion (Figure 4B), confirming the expression profile observed in sRNA libraries analyses (Figure 4A). This result could suggest that this miRNA acts in the early stage of drought stress response, as has been demonstrated in another sugarcane genotype [36]. MiR168 was also down-regulated in the sRNA libraries analysis (Figure 4A); nevertheless, this miRNA was down-regulated only in one biological replicates of each cultivar (Figure 4B). These differences of miRNAs regulation observed between biological replicates and the profiles obtained in the sRNA libraries can be reflecting the fact that each miRNA is regulating genes involved in quite diverse biological phenomena. For instance, while some miRNAs are regulating leaf morphogenesis, [39], [40], plants can trigger different physiological mechanism in response to water stress [37], such as the leaf curling, leaf wilting and reduced leaf length [38]. The other miRNA analyzed - the miR397 - was repressed in response to water stress in sensitive cultivars and induced in tolerant cultivars (Figure 4A). The result of qRT-PCR (Figure 4B) showed a repression of this miRNA in sensitive cultivar from Rep1 and Rep2 (p-value <0.05), similar to the sequencing analysis. In contrast, miR397II shows a tendency to increase its expression in SP832847 and SP835073, confirming the profile observed in the sRNA libraries of tolerant cultivars.

In addition, we also verified the expression of four other miRNA (miR156V, miR167 VII, miR169 III and miR159 II) by qRT-PCR using samples from Rep1 (File S5). These four miRNA were induced in tolerant genotype SP832847 exposed to drought stress; however, only for miR156 V this up-regulation was statistically significant. The expression of miR159II was inverse to what was observed in the sRNA libraries. On the other hand, the profiles of miRNA expression found in pooled samples (tolerant and sensitive libraries) were validated for other miRNA (miR156 V, miR167 VII and miR169 III) in the qRT-PCR experiments.

**Figure 5:**
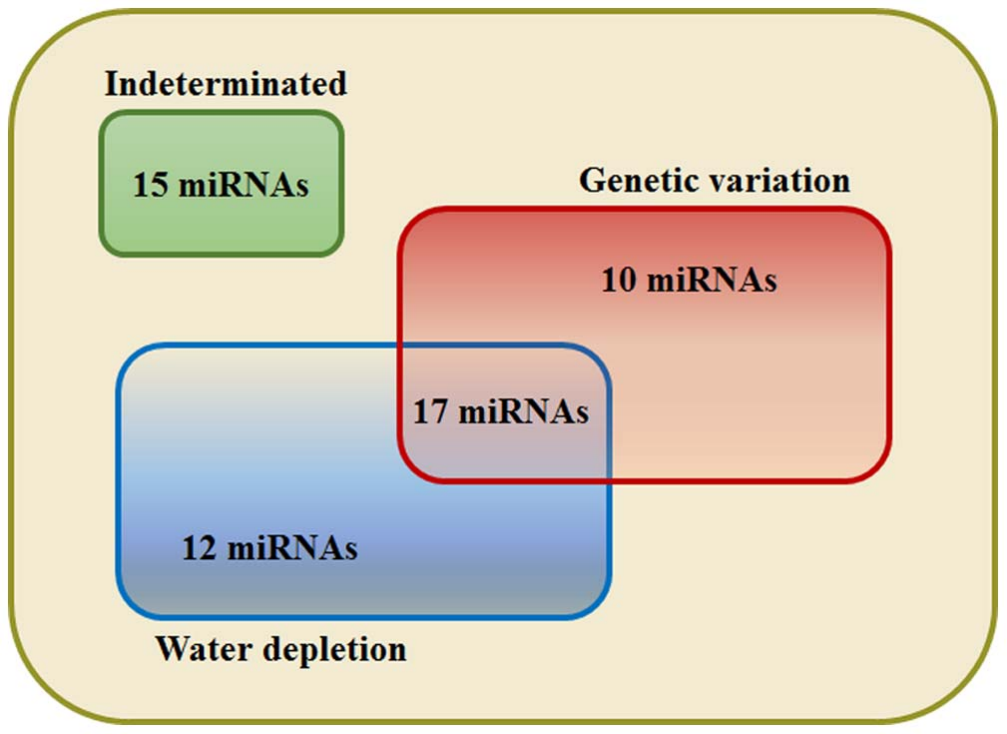
Regulation of miRNAs whether by genotype or/and water depletion. Comparison of miRNA regulation identified in individual cultivar libraries and pool libraries.

To verify whether the regulation of miRNAs observed in the pool libraries is mostly due to water depletion or could be influenced by genetic variation, two leaf libraries were constructed and sequenced using isolated sugarcane cultivars submitted to treatment of water depletion. These two cultivars, CTC6 and SP90–3414, were also used in pool libraries analysis, and according to the CTC, they have contrasting drought-tolerance. The identification of miRNA from these libraries was performed using a similar pipeline previously described for the pooled samples libraries, and the normalized abundance distribution of miRNAs identified is available in File S1. A total of 54 miRNAs had their expression profile compared in two conditions: comparison 1- to verify if miRNA regulation was influenced by genetic variation in response to stress treatment (T24h/S24h vs. CTC6/SP903414); and comparison 2– to verify if miRNA regulation was influenced by water depletion on tolerant and sensitive genotypes (CTC6/T0h vs. T24h/T0h) and (SP903414/S0h vs. S24h/S0h). These analyses resulted in a list of 39 miRNA that are positive for at least one of these comparisons (Table 2). Seventeen miRNAs had the same regulation profile in pool and individual cultivar libraries (Figure 5). Our results showed that 10 miRNAs share the similar expression profile only in comparison 1, suggesting an influence of genetic variation in miRNA response (Figure 5). In contrast, expression of 12 miRNAs was only influenced by water depletion (Figure 5). Among the miRNAs used for qRT-PCR, miR159 X and miR168 II had the same profile in both comparisons, suggesting that the of these miRNA regulation were influenced by both genotype and water depletion. These comparisons suggested that the regulation of miR164 II and miR397II was influenced by the treatment. Finally, for 15 miRNAs it was not possible to conclude if their regulation is mostly influenced by water depletion or by genetic variation (Figure 5).

**Table 2:** Comparison between miRNAs profile of pool plants and individual cultivar libraries.+ =  the miRNA profile is the same; - contrasting miRNA profile.

miRNA	Sequence	Comparison 1^a^	Comparison 2^b^
miR1439 I	ATTTGGAACGGAGGGAGTACT	**+**	**+**
miR156 I	GACAGAAGAGAGTGAGCACA	**+**	**+**
miR156 II	TGACAGAAGAGAGCGAGCAC	**+**	**−**
miR156 VI	TTGACAGAAGAGAGCGAGCAC	**−**	**+**
miR159 II	GGATTGAAGGGAGCTCTG	**−**	**+**
miR159 III	GTTGGATTGAAGGGAGCTCTG	**+**	**−**
miR159 V	TCTTTGGATTGAAGGGAGCTCTG	**−**	**+**
miR159 VII	TGGATTGAAGGGAGCTCTG	**+**	**+**
miR159 X	TTGGATTGAAGGGAGCTCTG	**+**	**+**
miR159 XI	TTGGATTGAAGGGAGCTCTGC	**+**	**+**
miR159 XIII	TTTGGATTGAAGGGAGCT	**+**	**−**
miR159 XVI	TTTGGATTGAAGGGAGCTCTG	**+**	**+**
miR159 XVII	TTTGGATTGAAGGGAGCTCTGC	**+**	**−**
miR162* I	GGGCGCAGTGGTTTATCGATC	**−**	**+**
miR164 I	TGGAGAAGCAGGGCACGTGCA	**+**	**+**
miR164 II	TGGAGAAGCAGGGCACGTGCT	**−**	**+**
miR166 I	TCTCGGACCAGGCTTCATTCC	**−**	**+**
miR166 II	TTCGGACCAGGCTTCATTCCC	**+**	**+**
miR166* I	AATGTTGTCTGGCTCGAGGTG	**+**	**−**
miR166* III	GAATGATGTCCGGTCCGAAGA	**+**	**+**
miR166* IV	GGAATGTTGTCTGGCTCGGGGG	**+**	**+**
miR167 III	TGAAGCTGCCAGCATGATCTA	**+**	**+**
miR167 IV	TGAAGCTGCCAGCATGATCTG	**+**	**+**
miR167 V	TGAAGCTGCCAGCATGATCTGA	**+**	**−**
miR167 VII	TGAAGCTGCCAGCATGATCTGG	**−**	**+** c
miR168 I	CCCGCCTTGCACCAAGTGAAT	**−**	**+**
miR168 II	TCGCTTGGTGCAGATCGGGAC	**+**	**+**
miR169 III	CAGCCAAGGATGACTTGCCGG	**−**	**+**
miR172 I	AGAATCTTGATGATGCTGCAT	**+**	**−**
miR1878 I	ATTTGTAGTGTTCAGATTGAGTTT	**+**	**−**
miR390 I	AAGCTCAGGAGGGATAGCGCC	**+**	**+**
miR390 II	AGCTCAGGAGGGATAGCGCC	**−**	**+**
miR395 III	TGAAGTGTTTGGGGGAACTC	**+**	**+**
miR396 II	TCCACAGGCTTTCTTGAACTG	**+**	**+**
miR397 II	TTGAGTGCAGCGTTGATGAGC	**−**	**+** c
miR398 II	TGTGTTCTCAGGTCGCCCCCG	**+**	**+**
miR444 II	TGCAGTTGTTGCCTCAAGCTT	**+**	**−**
miR528 I	TGGAAGGGGCATGCAGAGGAG	**+**	**−**
miR529 I	AGAAGAGAGAGAGTACAGCCT	**−**	**+**

a Comparison 1: influence of genetic variation on miRNA response to water depletion (T24h/S24h vs. CTC6/SP903414).

b Comparison 2: influence of water depletion treatment in miRNA response on each genotype (CTC6/T0h vs. T24h/T0h) and (SP903414/S0h vs. S24h/S0h).

c occur only in sensitive cultivars.

### Target Regulation

To achieve a better understanding of the biological function of the identified miRNA, we searched for putative miRNA targets using a bioinformatics approach. Based on perfect or near-perfect match between miRNA/target in plants [37], we used the plant target prediction tools available in a public website (http://srna-tools.cmp.uea.ac.uk) to identify targets for those miRNA identified. We searched for miRNA targets in the *Saccharum officinarum* ESTs – DFCI gene index v.3 sugarcane database. This analysis identified 677 putative targets for miRNA found in leaf libraries (File S6). The annotation of the miRNA targets shows that several of them are transcription factors. Interestingly, putative targets of some miRNA* were also identified (miR160* I, miR160* II, miR160* III, miR166* II, miR166* III, miR169* I, miR396* I and miR399* I).

**Figure 6:**
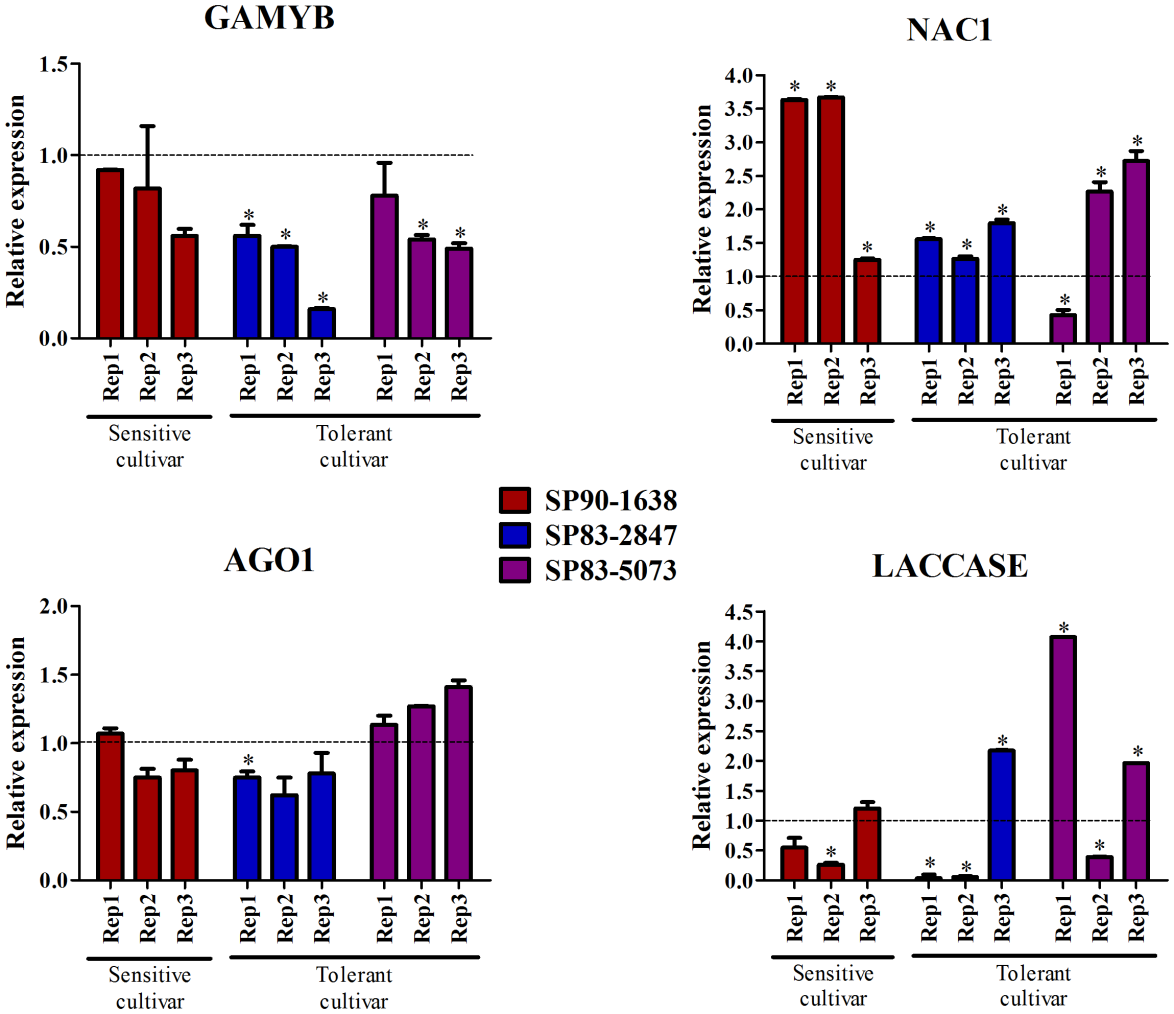
Relative expression profile of miRNA targets. Analysis of relative expression of miR159 target (GAMYB), miR164 target (NAC1), miR168 target (AGO1) and miR397 target (laccase) by qRT-PCR using samples of three sugarcane cultivar submitted to water depletion. In each case, control condition had relative expression equal 1 (dotted line). *represent significantly changing of miRNA expression between control and treatment samples (p-value <0.05).

Targets from some of the miRNA whose expression was validated were selected to confirm their regulation. The expression of GAMyb (target of miR159 X), NAC1 (target of miR164 I), AGO1 (target of miR168 II) and Laccase (target of miR397) was verified by qRT-PCR, using the same cDNA samples used for the analysis of mature miRNA expression. The results showed a negative correlation among the expression patterns of some these possible target/miRNA pairs (Figure 6). For instance, GAMyb was significantly repressed, around 50% or more, in the SP832847 tolerant cultivar submitted to water depletion treatment in three biological replicates, while the miR159X was induced in these same samples. The other pair that showed an inverse expression pattern of the correspondent miRNA was the miR164II/NAC1 transcription factor. NAC1 was up-regulated and miR164II was down-regulated in a drought-stress sensitive cultivar (p-value <0.05) in Rep1 and Rep2 samples. The NAC1 gene was induced (about 50%) while the miR164II was repressed (about 40%) in a tolerant cultivars, indicating a possible regulation of the NAC1 transcription factor by the miRNA. However, in the Rep3 of SP835073, we observed a repression of the target (p-value <0.05) although the expression of miRNA164II under water depletion did not change. In this case, the target was most likely regulated not only by miRNA164II. Alternatively, the miRNA could be induced earlier, reflecting in the late repression of target at 24 hs of treatment of water depletion. For miR168II and miR397II, their putative targets also did not show an inverse regulation (Figure 6).

### 22-nt miRNA Trigger siRNA Production in Leaves of Plants Under Stress

Recent studies revealed that 22-nt miRNA triggers the production of secondary siRNA [38], [39]. In order to identify siRNA regulated in sugarcane submitted to water depletion, we first identified putative targets of miRNA of 22 nt (Table 3). Next, a BLASTN was performed using the selected targets against the fraction of sRNA present in all libraries, excluding the list of miRNA sequences. A list of siRNA candidate sequences that have more than 50 reads in each library with their abundance is available in the File S7. The putative targets of miR319I, miR167V and three putative targets of miR159I probably originated siRNAs (Table 4). Sequences of siRNA from these miRNA targets varied 1–2 nt in 5′- or 3′- end of the sequence, but the most representative sequence in each case is highlighted in Table 4. Two miR159I-targets (CA229394 and TC134732) give rise the same siRNA candidates sequence. Moreover, the position of miRNA cleavage site in the target is the same of siRNA candidate, and these siRNA sequences are present in all libraries.

**Table 3:** 22-nt miRNA and their target prediction.

miRNA ID	Target gene accession	Start-end positionof target^a^	Target description
miR159I	CA229394	139–157	similar to UniRef100_A3LXX5 Cluster: Predicted mutarotase
miR159I	TC134732	551–568	similar to UniRef100_Q96464 Cluster: GAMyb protein
miR159I	CA185455	320–339	similar to UniRef100_A8TZU8 Cluster: AsmA
miR159XII	TC152911	775–795	similar to UniRef100_Q0DUE9 Cluster: Os03g0189900 protein
miR159XVII	CA229394	137–157	similar to UniRef100_A3LXX5 Cluster: Predicted mutarotase
miR159XVII	TC126115	268–290	similar to UniRef100_A7NXD7 Cluster: Chromosome chr5 scaffold_2, whole genome shotgun sequence
miR160*I	CA181132	416–433	
miR166*IV	No target found		
miR167I	TC137034	433–454	similar to UniRef100_Q0DLC3 Cluster: Os05g0109600 protein
miR167V	CA232593	420–439	similar to UniRef100_Q653U3 Cluster: Auxin response factor 17
miR167V	CA201181	176–195	homologue to UniRef100_A2YG67 Cluster: Auxin response factor 17
miR167V	CA292372	75–94	homologue to UniRef100_A2YG67 Cluster: Auxin response factor 17
miR167V	TC132917	399–418	similar to UniRef100_Q653U3 Cluster: Auxin response factor 17
miR167V	TC116192	466–485	similar to UniRef100_Q653U3 Cluster: Auxin response factor 17
miR167V	TC134152	357–376	homologue to UniRef100_Q653U3 Cluster: Auxin response factor 17
miR167V	TC114828	576–595	similar to UniRef100_Q6L8U0 Cluster: Auxin response factor 4
miR167V	TC151621	670–689	similar to UniRef100_A7Q186 Cluster: Chromosome chr10 scaffold_43, whole genome shotgun sequence
miR167V	TC137034	433–454	similar to UniRef100_Q0DLC3 Cluster: Os05g0109600 protein
miR167VII	TC137034	432–454	similar to UniRef100_Q0DLC3 Cluster: Os05g0109600 protein
miR319I	TC153622	223–241	similar to UniRef100_Q0D8K4 Cluster: Os07g0152000 protein
miR319I	TC115735	883–902	homologue to UniRef100_Q9M7E5 Cluster: Elongation factor 1-alpha
miR398I	TC112854	935–956	homologue to UniRef100_Q94KS7 Cluster: Beta-expansin 7 precursor
miR398I	TC136123	131–150	similar to UniRef100_A7Q737 Cluster: Chromosome chr5 scaffold_58, whole genome shotgun sequence
miR398I	TC120003	173–192	similar to UniRef100_Q93WS1 Cluster: Selenium binding protein
miR398I	CA124904	137–156	similar to UniRef100_A7Q737 Cluster: Chromosome chr5 scaffold_58, whole genome shotgun sequence
miR398I	CA233608	219–238	similar to UniRef100_Q1M2Z3 Cluster: Selenium binding protein
miR398I	TC136146	180–199	similar to UniRef100_A7Q737 Cluster: Chromosome chr5 scaffold_58, whole genome shotgun sequence
miR398I	CA170115	320–339	similar to UniRef100_Q6ZXK6 Cluster: Kelch repeat containing protein
miR398I	TC131025	327–346	similar to UniRef100_Q6ZXK6 Cluster: Kelch repeat containing protein
miR398I	CA124259	329–348	similar to UniRef100_Q6ZXK6 Cluster: Kelch repeat containing protein
miR398I	CA196471	318–337	similar to UniRef100_Q6ZXK6 Cluster: Kelch repeat containing protein
miR398I	CA231219	160–179	similar to UniRef100_Q6ZXK6 Cluster: Kelch repeat containing protein
miR398I	CA155584	568–589	–

a start and end position of miRNA and target alignment.

**Table 4:** miRNA target that originate siRNA candidates.

miRNA ID	Target	Cleavage site^a^	Library with siRNA^b^	Alignment position of siRNA^c^
miR159I	CA229394	139–157	T0h, T24h, S0h e S24h	157–139/294–311
miR159I	TC134732	551–568	T0h, T24h, S0h e S24h	568–551
miR159I	CA185455	320–339	S24h	495–475
miR167V	TC116192	466–485	T24h	755–773
miR319I	TC115735	883–902	S0h	115–137

a start and end position of miRNA and target alignment.

b libraries where found siRNA candidates. T = tolerant cultivar; S = sensitive cultivar.

c position that originate siRNA.

**Figure 7:**
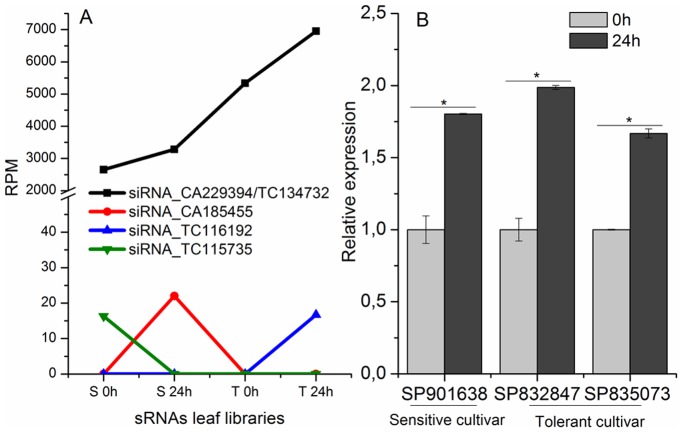
Analysis of siRNA candidates. Line graph is the abundance of siRNA candidates in each sRNA library normalized in reads *per* million. Bar graph is the relative expression of siRNA_CA229394/TC134732, which appeared in the four libraries, in different sugarcane cultivar submitted to water depletion by qRT-PCR. *represent significantly changing of miRNA expression between analyzed samples (p-value <0.05).

The abundance of the siRNA candidates in each library was calculated counting the number of reads of each exclusive sequence. To compare libraries, we normalized these values in transcript (reads) *per* million, using total number of reads in each library. Because siRNAs generated by the CA229394 and TC134732 resulted in identical sequences of siRNA, we combined these two targets. Figure 7A shows the number of normalized reads of siRNA candidates in each library. Only the siRNA_CA229394/TC134732 was presented in the four libraries, being the most abundant siRNA candidate identified. The others candidates were expressed only in one library. The expression of siRNA_CA229394/TC134732 was verified by qRT-PCR using the Pulsed qRT-PCR. The expression profile is similar to the observed in the bioinformatics analysis (Figure 7). This siRNA was significantly up-regulated in all genotypes when plants were submitted to water depletion during 24 h (Figure 7B), indicating a response to drought stress independent of the genotype of the cultivar.

### Water Depletion-affected siRNA Loci Regulation in Tolerant Cultivars

**Figure 8:**
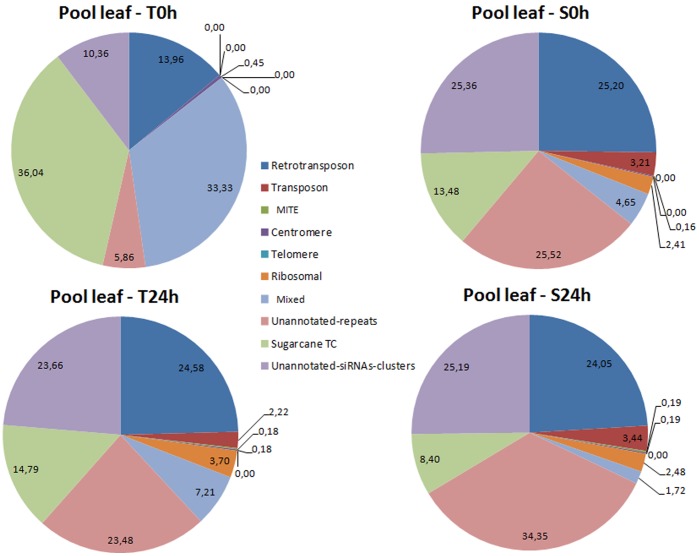
Distribution of leaf siRNA candidates aligned in cluster of siRNA. Each graph represents one leaf sRNA library. T = tolerant sugarcane cultivar, S = sensitive sugarcane cultivar. 0 h = control of stress, 24 h = time of water depletion treatment.

For further investigate the regulation of siRNA by water depletion in sugarcane, the fraction of sRNA present in all libraries, excluding the miRNA sequences, were aligned in clusters of siRNA. The clusters were constructed using a dataset of small RNA libraries consisting of 20 libraries (with miRNA sequences excluded). Next, the clusters of siRNA were annotated using information from a sugarcane repeat database (Grativol *et* al., in preparation) and in the sugarcane EST databases. The results showed a unique profile in the T0h leaf library (Figure 8) with more reads matching the Tentative Consensus (TC) EST (Expressed Sequence Tag) assembled and unclassified repeats than others libraries. Included are siRNA clusters that aligned with repeats that could be assigned to a repeat class (e.g. Retrotransposon, MITE) in Plant Repeat Database. Moreover, this library showed less reads, which aligned with retrotransposons, unannotated repeats and unannotated-siRNA-clusters. Interestingly, the profile of siRNAs aligned in other three leaf libraries is similar. Among the alterations in the retrotransposon families, the major change was in the LTR-gypsy. Stressed tolerant cultivars showed an increase of siRNA aligned with LTR-gypsy. Moreover, libraries of tolerant cultivar under water depletion (T24h) and sensitive cultivar control (S0h) are more similar. In all libraries, the fractions with less aligned reads were transposons, MITEs (miniature inverted-repeat transposable elements), centromere, telomere and ribosomal sequences (Figure 8). The classification of unannotated repeat refers to siRNA clusters that matched to unannotated repeats in the sugarcane repeats database. Unannotated-siRNA-clusters did not have matches with sugarcane repeats databases, but based on its abundance it is a putative siRNA cluster. We analyzed this distribution in root libraries and similar profiles were observed in the four libraries (File S8).

**Figure 9:**
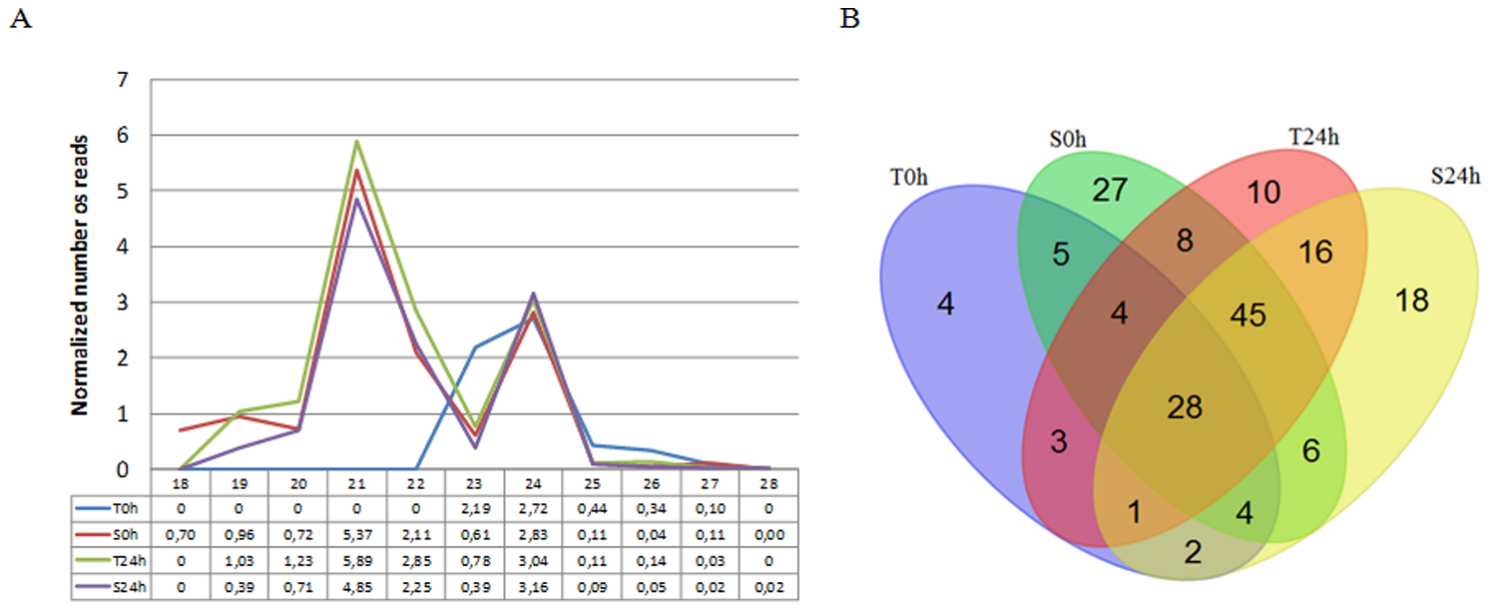
Analysis of siRNA candidates that aligned in siRNA cluster. (A) Length distribution of siRNA in each leaf library. (B) Venn diagram comprising the siRNA distribution in each leaf library. T = tolerant sugarcane cultivar, S = sensitive sugarcane cultivar. 0 h = control of stress, 24 h = time of water depletion treatment.

The size distribution of siRNA candidates in leaf libraries showed that the T0h sample showed a profile unlike the other libraries (Figure 9). It does not contain 21 nt reads and it has an additional peak of 23 nt. In the other three libraries, we observed two peaks, 21 nt (highest peak) and 24 nt. Next, we investigated the distribution of siRNA candidate sequences in the sRNA libraries (Figure 9 B). The result showed that 28 siRNA candidates were shared between leaf libraries. The S0h library showed the highest number of expressed siRNA candidates. Sensitive cultivars showed a higher number of exclusive siRNA candidates, while the tolerant cultivars showed a lower number of siRNA candidates. In addition, we observed a high number (45) of siRNA candidates in common between S0h, T24h and S24h.

### Core Components of sRNA Biogenesis are Regulated in Response to Water Depletion

**Figure 10:**
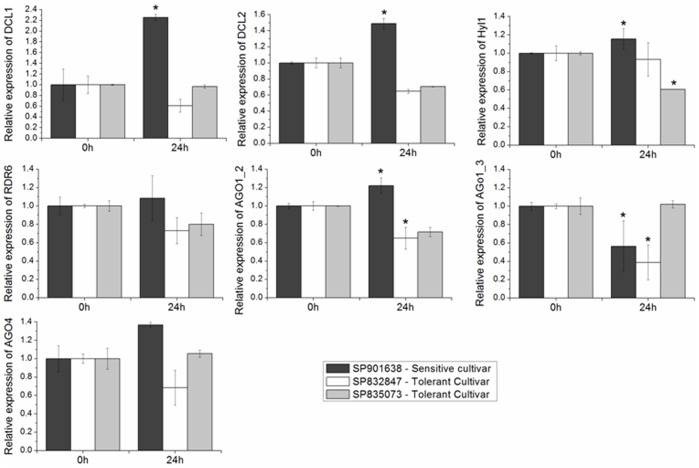
Relative expression of sRNA pathway genes. Analysis by qRT-PCR using samples of three sugarcane cultivar submitted to water depletion. *represent significantly changing of miRNA expression between analyzed samples (p-value <0.05).

To investigate whether the mechanisms of sRNA biogenesis could be altered in the libraries, we selected enzymes that participate in the pathways responsible for sRNA production. The biosynthesis of plant sRNA starts with the process of double strand RNA precursor. In plants, the precursor interacts with DICER-LIKE (DCL) endonucleases, which catalyzes excision of mature miRNA [16]. Four DCL have been identified in Arabidopsis [40], and we selected two sugarcane DCLs homologues to verify whether their expression profiles in sugarcane leaf submitted to water depletion were altered: DCL1 and DCL2 (Figure 10). Both DCLs are significantly induced in sensitive cultivar submitted to water depletion and this profile was not observed in the tolerant cultivars. These results suggest that the biogenesis of sRNA is increased in the sensitive cultivar. Accordingly, we have identified more miRNA in sensitive cultivars than in the tolerant cultivars. In addition, we verified the expression profile of HYPONASTIC LEAVES (HYL) 1, an enzyme in the miRNA biogenesis pathway [41]. The result showed a down-regulation only in one tolerant cultivar: SP835073 (p-value <0.05). In the sensitive cultivar, HYL1 was significantly up-regulated by water depletion. Plants encode RNA-dependent RNA polymerases (RDRs) to produce dsRNA which will give rise to siRNA [42]. Therefore, we analyze the expression profile of RDR6, and the results showed a small reduction in RDR6 expression in the tolerant cultivars after the stress. Finally, we analyze the expression of sugarcane genes AGO proteins. Arabidopsis has 10 genes encoding AGO proteins [43], [44]. In sugarcane, we identified four genes that are homologous to AtAGO1 and one homologous to AtAGO4. We investigated the expression of two of the AGO1 genes (AGO1_2 and AGO1_3) and AGO4 (Figure 10). The results showed that AGO1_ 2 was repressed in tolerant cultivars (about 40%); however, it was slightly induced in the sensitive cultivar (p-value <0.05). AGO1_3 was significantly down-regulated in the sensitive cultivar, but it showed different regulation patterns in the tolerant cultivars (SP832847– repressed; SP835073– unalterable). Likewise, AGO4 expression showed a tendency of repression in the cultivar SP832847, while the expression of AGO4 in the cultivar SP835073 did not vary. These results suggest that AGO4 expression increases in the sugarcane sensitive cultivar upon water depletion.

## Discussion

Plants are sessile organism and under natural conditions, they are often exposed to stresses caused by the environment. To respond and adapt to stress, plant have developed multiples regulatory mechanisms [45]. Recent studies have shown that many genes are epigenetically regulated in plants [46], [47]. Among the epigenetic modifications, DNA methylation is the most well-studied; and sRNA production is a novel epigenetic system that recently has been under intense investigation [6]. High-throughput sRNA sequencing has helped to accelerate the discovery of sRNA. In the present study, we have used deep sequencing to identify sRNA regulated in sugarcane plants submitted to water depletion. Drought stress is one of the major constraints to agricultural productivity worldwide and recent reports have highlighted the importance of sRNA in the response and adaptation to water availability to plants [48]. This study investigated the role of two classes of sRNA, the miRNA and the siRNA in the early responses of sugarcane to drought stress. First, we constructed and sequenced sRNA libraries from roots and leaves of sugarcane genotypes with different drought sensitivities. Consistent with most recent reports [49], [50], roots libraries had two major peaks of sRNA of 21 and 24 nt (Figure 2). Unexpectedly, a peak of sRNA of 22 nt in length was observed in leaf libraries. In a recent studies a peak of 22 nt was also observed in the size distribution of *Cucumis sativus* sRNA [51], [52].

Recent reports have revealed that plant miRNA are responsive to abiotic stress such as drought, cold, heat and salinity [48]. The goals of this work were to identify miRNAs present in sugarcane, and investigate their regulation in response to water depletion in different genotypes. Based on characteristics of plant miRNA conservation [24], [25], all plant miRNA deposited on the miRBase were used to identify sugarcane miRNA. Bioinformatics analysis identified 88 conserved miRNA or miRNA* in leaves and 273 in roots, being 64 sequences in common between the two sugarcane tissues. MiR159 is the more representative miRNA family in all cultivars, in both tissues. However, analysis of miRNA expression revealed that many of them are expressed only in certain tissues and/or cell specific, and at stage of development [53]–[55]. This could explain the difference in miRNA abundances found in roots and leaves of sugarcane. DCL1, the enzyme that cleaves the miRNA precursor in plants, releases preferentially 21-nt shorts RNA from dsRNA [56]; and immunoprecipitation with anti-AGO1 antibodies revealed the preferential association of AGO1 (the canonical enzyme of miRNA pathway) with sRNA containing 5′-terminal U [12]. Accordingly, the majority of the sugarcane miRNA identified were 21 nt in length and have uracil (U) at the 5′-end in both tissues. Furthermore, although miRNA* are normally less abundant [48], some studies have indicated that plant miRNA* could play a role in plant stress tolerance [57], [58]. In this study, 11 and 54 miRNA* were identified in leaf and in root libraries, respectively, and putative targets for leaf miRNA* were found. MiR169* had a larger number of targets, and most of them encode Elongation factor 1-alpha (EF 1-α). Accordingly, the role of EF 1-α has been analyzed in response to abiotic stress, including in response to water deficit [59]. Others miRNA* also have putative targets related to abiotic stresses, such as Kelch repeat-containing F-box like protein (target of miR396*) and cold induced protein like (target of miR399*).

Recent studies have shown that some miRNA are responsive to drought stress. In sugarcane, it has also been shown that miRNA are differently expressed in sRNA libraries of cultivars with contrasting tolerance to drought [27], [36]. MiR156, miR159, miR164, miR167, miR168, miR168 and miR397 are examples of miRNA that have been described to be regulated under drought stress conditions [60]–[62]. In this study, we investigated whether these miRNA were also regulated by water depletion in sugarcane. The qRT-PCR results confirmed the majority of the expression profiles observed in the bioinformatics analysis; however, there were a few that were not confirmed and this lack of correlation could be explained because a pool of genotypes was used in the construction of sRNA libraries, while in the qRT-PCR analyzes we examined miRNA expression in individual genotypes. In order to verify whether these microRNAs are differentially regulated by water depletion or by the genotype, two sRNA libraries of individual cultivars submitted to water depletion (one tolerant and one sensitive) were sequenced. In both rice [63] and sugarcane [33] cultivars, differential miRNA in response to water deficiency be at least partially explained by genetic variation. Our results have shown that at least a fraction of the microRNAs regulated in the pool libraries have similar regulation in cultivars with contrasting tolerance to drought, suggesting an influence of the environment in miRNA regulation.

The biological function of miRNA can be inferred by the knowledge of their targets. Targets can be regulated by cleavage, repression of translation, or DNA and histone methylation [14], [64]. In plants, bioinformatics/*in silico* analysis of miRNA and their targets genes is possible due to the perfect or near-perfect complementarity between miRNA/target in the binding sites [37]. Several sugarcane miRNA targets are transcription factors, in agreement with previous reports on conserved miRNA targets [34], [65], [66] and some of these are involved in a signaling pathway of plant adaptation to drought stress [67]. For instance, the transcription factor GAMyb was identified as a miR159 X target. GAMyb is a plant growth regulator and its regulation has been described during abiotic stress [4], [34]. The target of miR164 I is NAC1 (No Apical Meristem -NAM, Arabidopsis transcription activation factor- ATAF, Cup-shaped cotyledon- CUC), a transcription factor that has been reported as involved in tolerance to abiotic stress [68]. In rice, the overexpression of NAC1 produced plants more tolerant to drought and salt stress [69]. The modulation of miRNA (up- or down-regulation) can result in a modification of their target abundance under stress condition [70], [71]. An inverse expression correlation of GAMyb and NAC1 and their respective miRNAs was confirmed for one tolerant and the sensitive cultivar. The differences in miRNA regulation observed between the two tolerant cultivars could be associated of their different-physiological response to the stress. Accordingly, the reduction in chlorophyll content in response to drought stress is higher in SP835073 than SP835073, indicating an increased sensitive to this stress [72]. Expression of others targets, Laccase (target of miR397 II) and AGO1 (target of miR168 II) was analyzed. In both cases, there is not an inverse correlation between target and miRNA, although, the predicted targets are canonical targets for their respectively miRNA. In this case, it is possible that the post-transcriptional regulation of the canonical target by miRNA-directed cleavage is not limiting the accumulation of mRNA under the stress condition, and another layer of regulation is acting, similarly to what has been described in other plants [73]. In addition, different cultivars develop contrasting morphological characteristics in response to water depletion that could result in differences in the miRNAs regulation. For example, although miR397 is similarly regulated in tolerant cultivars, its target, the enzyme laccase, which is involved in lignin biosynthesis [74], is highly induced in SP835073. This cultivar is classified as high fiber content, and upon water depletion, could increase cell wall biosynthesis to prevent water loss.

Another important role of miRNA is in the production of siRNA. The class of siRNA that is produced after cleavage of the target by miRNA is the *trans*-acting (ta-) siRNA [75], [76]. Recently, it has been described that miRNA of 22 nt in length also triggers secondary siRNA biogenesis [77]. Based on this observation, and in their abundance in leaf libraries, 22-nt miRNA species and their targets were selected to identify putative ta-siRNA. Several *TAS* genes processed by miRNA to generate ta-siRNA are well characterized in plants [75]. Examples of these genes are *TAS1*, *TAS2*, *TAS3* and *TAS4* that are transcripts recognized by miR173 (*TAS1* and *TAS2*), miR390 (*TAS3*) and miR828 (*TAS4*). However, a recent study has showed the cleavage of NB-LRR defense genes by miRNA produces ta-siRNA [39]. In addition, *MYB*-derived siRNAs were described, and the miRNA that regulated this gene was miR828 in apple [78]. In our analysis, the target of miR159I (encoding a Myb family protein) originated ta-siRNA candidates present in all libraries. Interestingly, this sugarcane siRNA was up-regulated in samples from water depletion treatment, independent of the tolerance of the cultivar to stress, suggesting the involvement of this siRNA in the response to water depletion. The production of the discrete type of ta-siRNA (not in phase) described here could be a novel mechanism of sRNA-based regulation. Contrary to the canonical ta-siRNAs whose production silence additional targets, the sugarcane ta-siRNA matches the same gene as the original microRNA and could be a tool to reinforce the silencing. Nevertheless, this hypothesis needs experimental confirmation, once the failure to detect low abundance in-phase siRNA could be due to the lack of sugarcane genome information.

The population of sRNA is complex and in addition of the ta-siRNA class described above, a large portion of plants sRNA is originated from repeats [6]. This study revealed altered distributions of siRNA- derived from repeats and gene- among sugarcane cultivars with different tolerance of drought stress. The profile of siRNA clusters derived from TC EST observed in the sensitive cultivars without water depletion is similar to tolerant cultivars under treatment. Interestingly, tolerant cultivars without treatment showed more siRNA candidates from siRNA clusters, classified as TC EST, than the others libraries. Analyzing these TC ESTs, we observed that many are related to genes of photosystem I and II (data not shown). It is known that water depletion decreases the activities of photosynthetic carbon reduction cycle enzymes [79], and possibly this reduction could be the result in an increase in siRNA derived from these TC EST in sugarcane submitted to water depletion.

Different classes of sRNA are involved in changes in DNA methylation (24-nt species) and in the regulation after transcription (21-nt species) of TEs (transposable elements). In wheat, under normal conditions, most TEs families were regulated by sRNA with 24-nt [80]. In contrast, both sensitive and tolerant sugarcane genotypes under water depletion showed an increase of 21-nt species regulating retroelements. A recent study showed that drought stress induced changes in DNA methylation. MSAP analysis in rice revealed that the retrotransposon like LTR-gypsy was a polymorphic DNA methylated fragments in response to drought stress [81]. The wheat tolerant cultivar without stress showed a similar profile of 24-nt-based regulation (DNA methylation) of LTR-gypsy. Interestingly, in tolerant sugarcane cultivars, water depletion induced a shift in sized of LTR-gypsy derived siRNA from 24-nt to 21-nt species. These results suggest water deficiency may change methylated sites of TEs starting a sRNA-directed posttranscriptional silencing suppression of TE activity in sugarcane.

An important characteristic that differentiates sRNA classes (miRNA and siRNA) is their origin and biogenesis, even though some classes share common enzymes in their pathways [82]. DCL, HYL, RDR and AGO are examples of enzyme involved in the plant sRNA pathways. DCL1 is involved in the first step of canonical miRNA biogenesis and can be involved in production of miRNA-like siRNA and DCL1-dependent 22-nt siRNA; while, DCL2 is involved in cleavage of long inverted repeat precursor of miRNA-like siRNA [82]. The expression profile of these DCLs in sugarcane suggested a more effective activity of sRNA biogenesis in the sensitive than in tolerant sugarcane cultivars. The result is in agreement with the observation that more miRNA have been identified in sensitive cultivars than in tolerant cultivars. RDR6 is required for siRNA biogenesis, including ta-siRNA biosynthesis which convert the cleaved target transcript into double-strand RNA to be further processed by DCL [42], [83]. Differential expression of RDR6 was shown in sugarcane cultivars with contrasting tolerance to drought stress, with RDR6 expression being down-regulated in the tolerant cultivars. The first steps in miRNA processing requires the DCL1-HYL1 complex [84]. However, HYL1 was down-regulated only in the SP835073 (tolerant cultivar); therefore, it was not possible to conclude whether regulation of HYL1 expression is involved in the plant responses to water depletion. The mature miRNA is incorporated in a ribonucleoprotein complex (RISC) containing the AGO protein [85]. Following the mechanism of sRNA action, the AGO1 can trigger the cleavage of targeted RNA or inhibit mRNA translation [12], [13]. The expression profile of three AGO1 genes (AGO1-miR168II target, AGO1_2 and AGO1_3) was analyzed. Still, AGO1_2 and AGO1_3 were down-regulated in tolerant cultivar. The expression of the AGO4 gene was not significantly different in sugarcane cultivars submitted to water depletion. AGO4 has affinity for miRNA or siRNA and trigger the cleavage of target or mediate cytosine DNA methylation [86]. Moreover, generally, AGO4 binds sRNA that has 5′ adenosine 24-nt [87]. Altogether, these results suggest that some components of sRNA biogenesis are involved in the plants responses to water depletion.

Our results characterized the regulation of sRNA and explored the expression profile of sRNA pathway genes in sugarcane cultivars submitted to water depletion. We confirm the findings of recent studies that correlate the response of miRNA and their targets genes to availability of water. Furthermore, we showed that sRNA responses to water depletion could differ between sugarcane cultivars, contributing to increase the knowledge of sRNA roles in response to drought stress in the complex, polyploidy sugarcane plant.

## Supporting Information

File S1 **Electronic Northern of leaf miRNA.** The number of conserved miRNA reads in each library were normalized by transcripts per million. The miRNA sequences and their length were also shown.
(XLSX)

File S2 **Electronic Northern of root miRNA.** The number of conserved miRNA reads in each library were normalized by transcripts per million. The miRNA sequences and their length were also shown.
(XLSX)

File S3 **Pairwise statistical analysis of leaf miRNAs.** Statistical analysis of bioinformatics data using pairwise Fisher exact test, p-value <0.05.
(XLSX)

File S4 **Pairwise statistical analysis of root miRNAs.** Statistical analysis of bioinformatics data using pairwise Fisher exact test, p-value <0.05.
(XLSX)

File S5 **Relative expression of miRNA in different sugarcane cultivars by qRT-PCR.** *represent significantly changing of miRNA expression between analyzed samples (p-value <0.05).
(XLSX)

File S6 **Putative targets of all leaf miRNA in sugarcane.** EST sugarcane data from Gene Index version 3.0 was used to search for potential new miRNA targets by UEA sRNA toolkit-Plant target prediction pipeline.
(XLSX)

File S7 **siRNA candidates from targets of miRNA 22-nt in each library.**
(XLSX)

File S8 **Distribution of root siRNA candidates aligned in cluster of siRNA.** Each graph represents one-leaf sRNA library. T = tolerant sugarcane cultivar, S = sensitive sugarcane cultivar. 0 h = control of stress, 24 h = time of water depletion treatment.
(XLSX)
